# Social poverty indicators with school bullying victimization: evidence from the global school-based student health survey (GSHS)

**DOI:** 10.1186/s12889-024-18119-3

**Published:** 2024-02-26

**Authors:** Lin Chen, Ying Chen, Hailiang Ran, Yusan Che, Die Fang, Qiongxian Li, Yuanyu Shi, Shuqing Liu, Yandie He, Guiqing Zheng, Yuanyuan Xiao

**Affiliations:** NHC Key Laboratory of Drug Addiction Medicine, Division of Epidemiology and Health Statistics, School of Public Health, Kunming Medical University, 1168 West Chunrong Road, Yuhua Street, Chenggong District, 650500 Kunming, Yunnan China

**Keywords:** Social poverty indicators, Bullying victimization, Correlational study, Children and adolescents

## Abstract

**Background:**

School bullying is prevalent in children and adolescents. Bullying victims are seen higher risk of negative psychological outcomes. Previously published studies suggested that social indicators may pose significant influence on bullying victimization. However, the association between social poverty and bullying victimization has not been exclusively discussed.

**Methods:**

In this cross-sectional study, we analyzed the association between 6 commonly used social poverty indicators (Poverty Headcount Ratio, PHR; Poverty Gap, PG; Squared Poverty Gap, SPG; monthly household per capita income, PCI; Watts’ Poverty Index, WPI; the Gini Index, Gini) and the prevalence of school bullying at country level by using the Global school-based Student Health Survey (GSHS) database.

**Results:**

Altogether 16 countries were included into the final analysis, with school bullying victimization prevalence ranged from 12.9 to 47.5%. Bubble plots revealed statistically significant associations between the three indicators measuring absolute poverty level (PHR, PCI, WPI) and bullying victimization. Subsequently performed principal component regression indicated that, for all types of bullying victimization, the increase of absolute poverty level was related to elevated prevalence rates, and the association was particularly strong for verbal bullying victimization.

**Conclusions:**

Our study results may suggest that absolute social poverty is an important parameter for constructing and implementing school bullying victimization intervention strategies and measures.

**Supplementary Information:**

The online version contains supplementary material available at 10.1186/s12889-024-18119-3.

## Background

Bullying is an aggressive act or social interaction in which an individual or group with a power advantage repeatedly humiliates or intimidates someone [1]. There are several types of bullying, traditional bullying (physical, verbal, and relational), and a new form of cyber bullying exerted via electronic media [2]. School bullying among children and adolescents is prevalent globally, with estimated prevalence surpassed 65% in some countries [3]. Both traditional and cyber bullying can impose detrimental influence on mental health of children and adolescents [4–6]. For instance, bullying can lead to increased risk of depression, anxiety, post-traumatic stress disorder (PTSD), interpersonal violence, and suicidal behaviors [7–10].

Children and adolescents can be implicated into school bullying as pure victims, pure bullies, or bully-victims [11]. It has been found that any role of school bullying involvement is associated with significantly impaired short-term and long-term mental health status, particularly for bullying victims [12]. Studies have disclosed that bullying victims were observed poorer academic performance, and increased risk of physical, emotional, and behavioral problems [13–14]. In addition, the risk of self-harm for bullying victims was about 6 times compared with people who were not involved in school bullying [15].

Searching for influencing factors of school bullying victimization is essential for developing effective prevention policies and measures. Many published studies have identified risk factors at individual level, such as body weight, physical disability, anxiety, depression, low self-esteem, etc. [16].. It has been found that social factors may also have a significant effect on bullying victimization. For instance, Liang et al. reported that adolescents in countries with food insecurity were more likely to be victims of bullying, with an odds ratio (OR) of 1.37 [17]. In addition, Deryol et al. observed that countries with high inequality-adjusted Human Development Index (HDI) had a lower prevalence of bullying victimization [18].

Social poverty, measured by indicators such as monthly household per capita income (PCI) and Poverty Gap (PG), reflect either socioeconomic status (absolute poverty) or inequality (relative poverty) of the society. It has been found that social poverty is significantly related to higher risk of cognitive development problems, social-emotional issues, anxiety, depression, and other mental health problems in children and adolescents [19–21]. Given the intimate relationship between mental health and school bullying behaviors in youths, it is reasonable to suspect a connection between social poverty and school bullying victimization. However, this hypothesis has not been effectively discussed.

In the current study, we intend to evaluate the association between social poverty indicators and prevalence of school bullying victimization at country level. The findings of our study are expected to help formulate effective regional or national school bullying victimization prevention and control initiatives.

## Data and methods

### Data sources

This study used data from the Global School-based Student Health Survey (GSHS), an ongoing multi-national survey program started in 2003. The GSHS was developed by the World Health Organization (WHO) in collaboration with several agencies in the United Nations. GSHS is a school-based survey conducted primarily among students aged 13–17 years. The questionnaire is designed with ten core modules: alcohol use, dietary behaviors, drug use, hygiene, mental health, physical activity, protective factors, sexual behaviors, tobacco use, violence and unintentional injury. Our study used publicly available data from 16 countries (Chile, Trinidad and Tobago, Uruguay, Argentina, Colombia, Costa Rica, Dominica, Ecuador, Guyana, Malaysia, Peru, Suriname, Belize, Bolivia, Honduras, El Salvador) [22]. Here in the current study, information regarding to bullying victimization was extracted from the “violence and unintentional injury” module. The data were retrieved from surveys conducted between 2013 and 2015, for countries with repeated measuring databases, we used the most recent one. For surveyed countries, nationally representative data will be the first choice, if nationally representative data are not available, then reginal data will be included. The GSHS surveys were approved by both a national government administrative body and an institutional review board or ethics committee in each country. Verbal or written informed consents were obtained from the participants and their parents. All social poverty indicators of the surveyed countries within the same time interval were retrieved from the World Bank [23, 24].

### Measurements

#### Bullying victimization

Bullying victimization was ascertained by using the question “During the past 30 days, on how many days were you bullied?”. Answers to this question include: 0 days, 1 or 2 days, 3 to 5 days, 6 to 9 days, 10 to 19 days, 20 to 29 days, all 30 days. Respondents who answered “1 or 2 days” or more frequently been bullied were classified as bullying victims.

#### Types of bullying victimization

Different types of bullying victimization were determined by using the question “During the past 30 days, how were you bullied most often?”. Answers to this question include: (A) I was not bullied during the past 30 days; (B) I was kicked, pushed, or shoved; (C) I was made fun of because of religion; (D) I was made fun of with sexual jokes; (E) I was left out of activities; (F) I was made fun of because of my body; (G) I was made fun of because of my race, nationality, or color; (H) I was bullied in some other way. Respondents who chose B, E, C/D/F/G were classified as victims of physical, relational, and verbal bullying, respectively.

#### Social poverty indicators

We used six social poverty indicators in this study, with a poverty line set at $1.90 per person per day [25]: Poverty Headcount Ratio (PHR), Poverty Gap (PG), Squared Poverty Gap (SPG), monthly household per capita income (PCI), Watts’ Poverty Index (WPI), and the GINI Index. PHR refers to the percentage of the population living below the national poverty line. PG is the proportion of the population with average income falls below the poverty line. SPG is calculated by averaging the square of PG. Monthly household PCI was ascertained in comparison to 2011 purchasing power parity (PPP). WPI is measured as the logarithm of the ratio of the poverty line to income. The Gini index measures deviations from a perfectly equal distribution of incomes within an economy among individuals or households, which ranges from 0 (no inequality) to 100 (totally unequal) [26–27].

### Statistical analysis

Descriptive statistics were used to illustrate bullying victimization prevalence and the six social poverty indicators for the 16 countries analyzed. The associations between poverty indicators and bullying victimization prevalence were detected by using bubble plot. Strength of the association was calculated, and the variables showing significant correlation (*p* < 0.05) were further analyzed by using multiple regression methods. Prior to multiple regression, considering the possible inter-correlations between the included social poverty indicators, to avoid collinearity, we used principal component analysis (PCA) to extract prominent factors, and then used principal component regression to estimate the adjusted association between social poverty components and bullying victimization. All analyses were performed using the R statistical software (Version 4.2.0, The R Foundation for Statistical Computing, Vienna, Austria). Statistical significance was set as a two-tailed probability no higher than 0.05.

## Results

### Descriptive statistics

Table 1 shows the overall prevalence rates together with 95% confidence intervals (CIs) of bullying victimization in general, and by different types, for each included country. Among the 16 countries, the prevalence of bullying victimization ranged from 12.9% (95% CI: 11.44-14.36%) to 47.5% (95% CI: 45.67-49.33%); the highest prevalence was in Peru (47.5%), whereas the lowest was in Chile (12.9%); for different types of bullying victimization, the highest and lowest prevalence of physical bullying were found in Dominica (4.6%) and Uruguay (0.8%), Peru (18.4%) and Trinidad and Tobago (6.8%) reported the highest and lowest prevalence of verbal bullying victimization, Peru (4.4%) and Suriname (0.5%) were found the highest and lowest prevalence of relational bullying victimization.

**Table 1:** Prevalence of bullying victimization in general and by different types for the 16 countries analyzed

Country	Bullying victimization, % (95% CI)	Physical, %	Verbal, %				Relational, %	Some other way, %
			Made fun of race	Made fun of religion	Made fun of sex	Made fun of body		
Chile	12.9 (11.44–14.36)	0.9	1.0	0.4	2.3	3.3	0.7	3.4
Trinidad and Tobago	16.7 (15.31–18.09)	3.1	2.1	0.8	1.4	2.5	0.6	4.0
Uruguay	18.6 (17.31–19.89)	0.8	1.0	0.2	2.9	4.9	1.1	5.6
Argentina	25.0 (24.49–25.51)	1.8	1.5	0.6	2.9	4.9	1.3	7.0
Colombia	30.9 (29.69–31.51)	2.3	1.7	0.7	3.0	5.3	3.0	14.6
Costa Rica	19.0 (17.51–20.49)	1.4	1.1	0.3	2.7	4.5	1.3	6.2
Dominica	26.1 (23.97–28.23)	4.6	2.1	0.9	2.7	4.4	1.0	8.0
Ecuador	27.7 (26.51–28.89)	3.9	3.2	1.4	2.9	3.0	2.0	11.3
Guyana	37.3 (35.35–39.25)	4.5	4.0	2.8	2.4	4.8	2.1	12.1
Malaysia	17.4 (16.93–17.87)	1.6	1.4	0.4	2.8	3.3	0.7	3.9
Peru	47.5 (45.67–49.33)	4.2	3.2	1.9	5.3	8.0	4.4	17.1
Suriname	27.0 (24.88–29.12)	1.0	1.7	0.8	1.6	3.8	0.5	14.5
Belize	30.3 (28.34–32.26)	4.3	3.1	1.2	2.1	5.3	1.6	10.1
Bolivia	32.4 (30.86–33.94)	3.3	2.6	1.7	2.8	3.6	1.7	9.0
Honduras	30.9 (28.73–33.07)	2.2	2.6	1.4	3.6	4.4	2.6	9.2
El Salvador	22.9 (21.00-24.80)	1.6	1.9	1.2	2.3	3.6	1.6	6.5

Table 2 summarized the associations between the six social poverty indicators. Generally, PCI was inversely associated with the other five indicators, a country with smaller PCI and larger PHR/PG/SPG/WPI/Gini were at higher level of social poverty. Among all the included countries, Suriname was at the highest level of poverty, followed by Honduras, and Malaysia had the lowest level of poverty.

**Table 2:** Six social poverty indicators for the 16 countries analyzed

Country	PHR	PCI	PG	SPG	WPI	Gini
Chile	0.36	667.07	0.16	0.11	0.16	45.83
Trinidad and Tobago	0.23	783.34	0.23	0.12	0.16	40.27
Uruguay	0.15	737.77	0.15	0.02	0.04	39.90
Argentina	1.16	616.23	1.16	0.26	0.47	41.33
Columbia	9.93	343.67	9.93	2.19	5.54	51.00
Costa Rica	2.44	625.92	2.44	0.73	1.22	50.56
Dominica	3.17	383.52	3.17	0.33	1.07	48.88
Ecuador	8.53	353.95	8.53	1.79	4.86	53.36
Guyana	8.96	294.22	8.96	3.40	11.65	45.12
Malaysia	0.06	710.34	0.06	0.00	0.01	43.90
Peru	5.54	350.06	5.54	0.66	2.14	45.55
Suriname	17.53	383.31	17.53	12.39	5.70	57.85
Belize	13.66	279.57	13.66	3.63	8.77	53.26
Bolivia	8.16	393.28	8.16	2.48	6.54	46.59
Honduras	19.00	238.70	19.00	4.28	12.17	53.37
El Salvador	3.36	303.76	3.36	0.27	0.96	43.36

PHR: Poverty Headcount Ratio; PG: Poverty Gap; SPG: Squared Poverty Gap; PCI: monthly household per capita income; WPI: Watts’ Poverty Index;

Gini: the Gini Index

### Social poverty indicators with bullying victimization

**Fig. 1:**
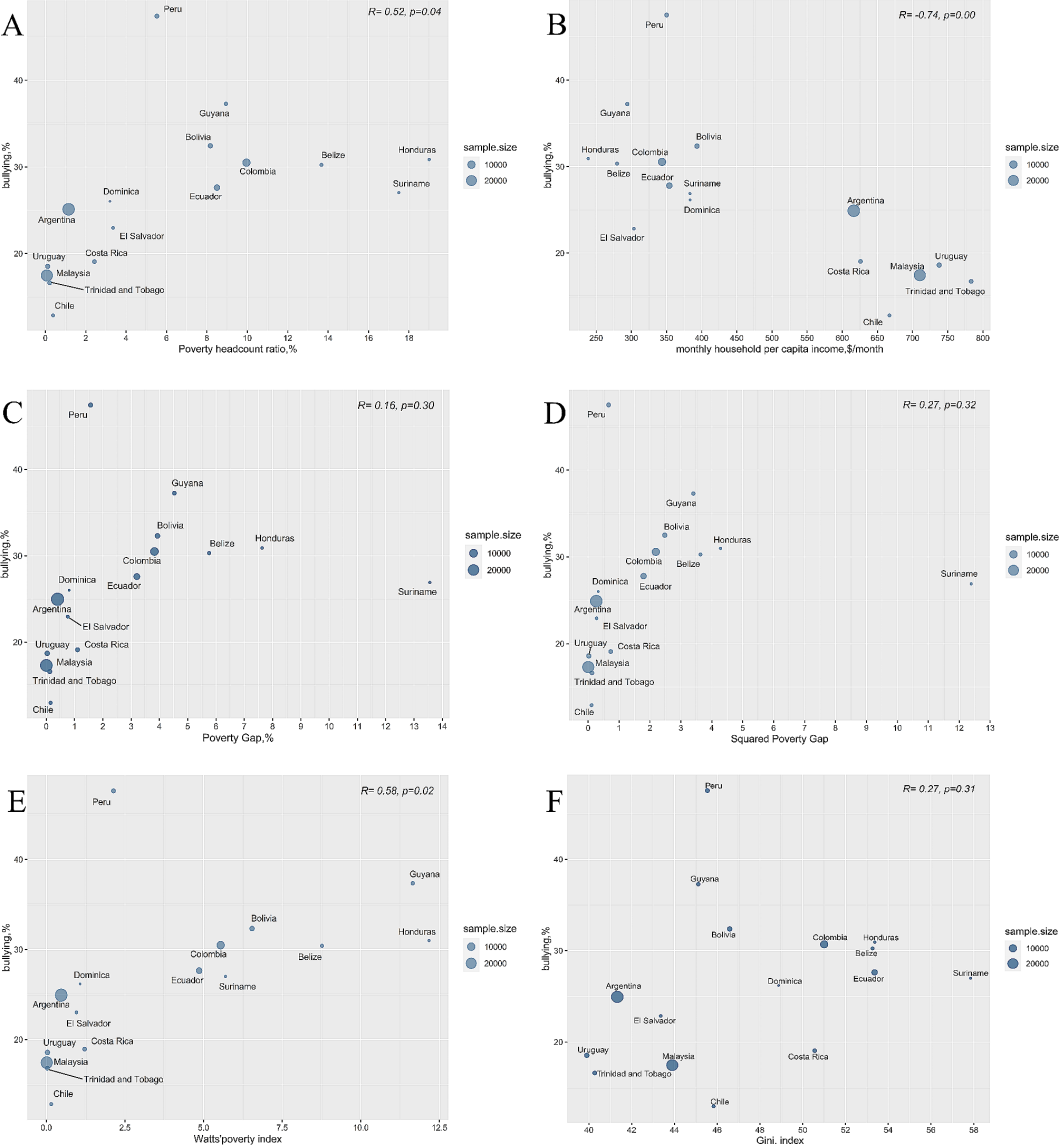
Bubble plots of associations between social poverty indicators and the prevalence of bullying victimization. (**A**): Poverty Headcount Ratio (PHR); (**B**): monthly household per capita income (PCI); (**C**): Poverty Gap (PG); (**D**): Squared Poverty Gap (SPG); (**E**): Watts’ Poverty Index (WPI); (**F**): the Gini Index (Gini)

Correlations between social poverty indicators and the prevalence of bullying victimization were estimated by using bubble plots, and the results were jointly illustrated in Fig. 1. As shown in the figure, PHR (*r* = 0.52, *p* < 0.05), PCI (*r*=-0.74, *p* < 0.05), and WPI (*r* = 0.58, *p* < 0.05) were significantly correlated with bullying victimization, whereas PG (*r* = 0.16, *p* > 0.05), SPG (*r* = 0.27, *p* > 0.05) and Gini (*r* = 0.27, *p* > 0.05) only showed statistically insignificant associations.

Multiple regression analysis was further conducted by including the three significant poverty indicators identified by bubble plots. As a high level of inter-correlation existed for the three poverty indicators (See in supplementary material, Table S1), principal component regression was used. By synthesizing the information from scree plot, eigenvalues, together with proportions of variance explained for extracted principal components (Table 3), only one principal component (PC1) had been extracted, its eigenvalue was 2.581, accounted for 86% of the total variance.

**Table 3:** Principal components and variances explained

	PC1	PC2	PC3
Eigenvalue	2.581	0.278	0.141
Proportion of variance	0.860	0.093	0.047
Cumulative proportion	0.860	0.953	1.000

### Principal component regression fitting results

We further calculated the value of PC1 by using the loading factors (the formula is: PC1 = 0.366*PHR + 0.363*WPI-0.349*PCI). Then, using PC1 as the comprehensive social poverty indicator, school bullying victimization prevalence as the dependent variable, we fitted a series of principal component regression models, and the fitting results were summarized in Table 4. PC1 showed statistically significant associations with the overall bullying victimization, as well as the specific types of bullying victimization. For the three types of bullying victimization, the comprehensive social poverty indicator PC1 presented the strongest association with verbal victimization (regression coefficient: 1.442, *p* < 0.05) followed by physical victimization (regression coefficient: 0.554, *p* < 0.05) and relational victimization (regression coefficient: 0.467, *p* < 0.05), all statistically significant.

**Table 4:** Fitting results for principal component regression models

Dependent	Regression coefficient of PC1	95% CI	p value
BV prevalence	5.755	3.30–8.93	< 0.05
Physical BV prevalence	0.554	0.05–1.37	< 0.05
Verbal BV prevalence	1.442	0.55–2.41	< 0.05
Relational BV prevalence	0.467	0.13–0.83	< 0.05
Some other way BV prevalence	2.784	1.46–4.78	< 0.05

BV: bullying victimization

## Discussion

In this study by using the GSHS database, as expected, we found a significant association between social poverty and school bullying victimization in children and adolescents at country level, with a higher level of social poverty related to increased prevalence of bullying victimization. However, among all the 6 social poverty indicators that we investigated, only PHR, PCI and WPI showed this significant association with bullying victimization. As for different types of bullying victimization, their associations with social poverty indicators differed, and the strongest association was seen for verbal bullying. The main findings of our study can provide valuable information for devising bullying victimization prevention and control strategies and measures in consideration of national poverty levels.

The positive association between social poverty status and school bullying victimization that we found can be well justified by existing literature. A previously published US study revealed that, youth with poverty-related problems of both food and housing insecurity were more than 3 times likely to report victimization, compared with youth without these adversities [28]. Moreover, based on the family investment model, impoverished families only have limited funds to invest in necessities of life for children, therefore children are more likely to be deprived [29–31]. Deprived children are seen higher risk of low self-esteem and being excluded by peers, which increased their chance of being bullied [32–34]. Besides, poverty leads to poor living conditions for adolescents, together with frequent move, family conflicts caused by economic pressures or inadequate awareness of family support, all of which can contribute to increased risk of bullying victimization [35–36].

An interesting finding of our study is that, for the 6 social poverty indicators that we analyzed, only the three indicators of absolute poverty (PHR, PCI, WPI) were significantly associated with bullying victimization, whereas the other three indicators measuring socioeconomic inequality (PG, SPG, GINI) showed insignificant impact. This finding is somewhat different from previous study by Fajnzylber et al., which found a clear association between inequality and violence, rather than poverty and violence [37]. More specifically with respect to bullying, some studies have found a relation between bullying and inequality, but not bullying and poverty, at country and school level [38–39], in contrast to the insignificant association between inequality and school bullying victimization that we found. One possible explanation to this heterogeneity could be that those studies primarily used subjective measures of inequality, compared with the objective inequality indicators that we used in the current study. Considering subjective inequality indicators are more informative than objective indices [40], it is possible that the association between inequality and bullying victimization was underestimated in the current study. Our findings suggest that, for countries characterized in absolute poverty, when constructing and implementing bullying intervention measures, children and adolescents who are living under the poverty line should be prioritized.

Another important finding to be noticed is that, for different types of bullying victimization, their associations with social poverty indicators varied. Verbal bullying victimization showed the strongest association with social poverty, followed by physical and relational bullying victimization. Verbal bullying is the most common type of traditional bullying [41]. According to literature, in children and adolescents, the prevalence of verbal bullying involvement can be as high as 53%, compared with 51% for relational bullying, and 21% for physical bullying [42]. Our study results suggest that this most common type of school bullying can be more significantly influenced by social poverty. Studies about natural or planned experiments in reducing poverty should be conducted to determine the effect on school bullying, especially on verbal bullying.

The current study is among the first attempts in estimating the association between social poverty and school bullying victimization at country level. Multinational representative survey data further consolidates the validity of our findings. Nevertheless, several limitations should be recognized. First, both the social poverty indicators and school bullying prevalence rates were measured at country level, therefore the essence of our study is ecological, which is prone to ecological bias. Future studies by using individual level data are warranted. Second, the GSHS is implemented in limited countries, and in this study, we only included countries with complete survey data in school bullying, therefore our results may suffer from selection bias. Third, because of data unavailability, other social indicators which may confound the association between social poverty and school bullying victimization could not be included and controlled for, therefore residual confounding may exist. Fourth, the sample size is small, and a larger sample size may yield more significant results. Fifth, the study sample do not include very rich or very poor countries, so it is possible that variance in poverty could be much larger with a larger sample size. Finally, comparing the level of school bullying depends on similar understanding of what the measures ask, however, it is difficult to translate the word “bullying” to different languages.

## Conclusions

In this correlational study, by using the GSHS database, we found a positive association between social poverty indicators and bullying victimization among children and adolescents at country level: a higher level of absolute social poverty was associated with increased prevalence of school bullying victimization, particularly for verbal bullying victimization. These major findings highlight the necessity of incorporating social poverty status when devising and implementing school bullying victimization intervention strategies and measures. Future studies with information measured at individual level are needed.

### Electronic supplementary material

Below is the link to the electronic supplementary material.

Supplementary Material 1

## Data Availability

The manuscript’s data are available from the corresponding author.

## Abbreviations

GSHS: the Global school-based Student Health Survey
PHR: Poverty Headcount Ratio
PG: Poverty Gap
SPG: Squared Poverty Gap
PCI: monthly household per capita income
WPI: Watts’ Poverty Index
Gini: the Gini Index
