# Supplementary material for: Social poverty indicators with school bullying victimization: evidence from the global school-based student health survey (GSHS)
Source: BMC Public Health. 2024 Feb 26;24:615. doi: 10.1186/s12889-024-18119-3 (PMC10898088; doi:10.1186/s12889-024-18119-3)
Supplement: Supplementary file 1 — Supplementary Material 1 [file 12889_2024_18119_MOESM1_ESM.docx]

Table S1 Correlation coefficients matrix of social poverty measures.

|  | PHR | WPI | PCI |
| --- | --- | --- | --- |
| PHR | 1 |  |  |
| WPI | 0.86^***^ | 1 |  |
| PCI | -0.77^***^ | -0.75^***^ | 1 |

^***^*p*<0.001
